# Superficial Fungal Infections in Children—What Do We Know?

**DOI:** 10.3390/jcm14207380

**Published:** 2025-10-18

**Authors:** Katarzyna Rychlik, Julia Sternicka-Rohde, Roman J. Nowicki, Leszek Bieniaszewski, Dorota Purzycka-Bohdan

**Affiliations:** 1 Department of Dermatology, Venereology and Allergology, Medical University of Gdańsk, 80-214 Gdańsk, Poland; katarzyna.rychlik@gumed.edu.pl (K.R.); julia.sternicka@gumed.edu.pl (J.S.-R.); roman.nowicki@gumed.edu.pl (R.J.N.); 2 Mycology Outpatient Clinic, University Clinical Centre, 80-214 Gdańsk, Poland; 3 Clinical Physiology Unit, Medical Simulation Centre, Medical University of Gdańsk, 80-210 Gdansk, Poland; leszek.bieniaszewski@gumed.edu.pl

**Keywords:** superficial fungal infections, fungi, mycoses, dermatophyte, pediatric, children, dermatology

## Abstract

Superficial fungal infections are common conditions affecting the skin, hair, and nails, primarily caused by dermatophytes, yeasts, and less frequently, molds. Humid climates, prolonged summer seasons, immunodeficiencies, diabetes and socioeconomic factors such as poor hygiene and overcrowding promote them. Children are particularly susceptible due to their immature immune systems and other contributing factors. The infections are classified based on the site involved and include, among others, scalp infections, athlete’s foot, or nail infections (onychomycosis). Scalp mycoses are primarily caused by dermatophytes of the genera *Trichophyton* and *Microsporum*, which may originate from human or animal sources. Onychomycosis is rare in young children, with *Trichophyton rubrum* and *Trichophyton mentagrophytes* being the most frequently isolated pathogens. The increasing incidence in pediatric populations is linked to atopy, immune disorders, and immunosuppressive therapies. Treatment involves topical and systemic medications, depending on the location and severity of the infection. Maintaining proper hygiene, addressing risk factors, and monitoring therapy are essential to prevent recurrence. Focusing on children, this review explores current epidemiological trends, diagnostic practices, and treatment options related to superficial fungal infections.

## 1. Introduction

Superficial fungal infections (SFIs) are common infections typically confined to the skin, hair, or nails. They rank among the leading dermatological conditions worldwide and are frequently caused by dermatophytes, yeasts, and, to a lesser extent, molds [1]. In recent years, the incidence of fungal infections in children has increased, driven by both primary factors—skin barrier dysfunctions such as atopic dermatitis, keratinization disorders, diabetes and immune system defects—as well as secondary factors, including the use of immunosuppressive drugs, antibiotics, organ transplantations, and HIV infection [2,3]. A variety of climatic, socioeconomic, and host-related factors can also contribute to the increasing incidence of fungal infections in children. Low socioeconomic status, poor hygiene, overcrowding, and inadequate healthcare, as well as environmental factors—longer summer seasons, higher humidity, and geographical location—increase the frequency of mycoses [2,3]. Fungal infections are highly contagious and are transmitted through direct skin contact, contaminated objects, and exposure to animals. They are often recurrent, and inadequate medical care facilitates the spread of localized epidemics. The increasing incidence of SFIs in children poses a serious public health challenge. This article aims to review current epidemiological trends, diagnostic approaches, and treatment strategies for superficial fungal infections in the pediatric population.

## 2. Etiological Factors

To date, approximately 40 species of dermatophytes have been identified, with about half responsible for the majority of human infections, affecting an estimated 20–25% of the global population. Historically, all pathogenic dermatophytes were classified into three genera: *Microsporum (M.)*, *Trichophyton*, *(T.)* and *Epidermophyton (E.)*. However, advancements in modern diagnostic methods have led to a revised taxonomy that now recognizes six genera, including three additional ones: *Nannizzia*, *Lophophyton*, and *Arthroderma* [4].

Dermatophytes are further classified according to their ecological niches into anthropophilic (e.g., *T. violaceum*, *T. rubrum*, *M. audouinii*), geophilic (e.g., *M. gypseum*, *M. nanum*), and zoophilic (e.g., *T. mentagrophytes*, *T. verrucosum*, *M. canis*) [5].

Anthropophilic species usually cause chronic infections with mild inflammation and low contagion potential, although transmission between humans is still common. They are primarily transmitted indirectly through contaminated objects such as hairbrushes or toys, although direct person-to-person contact contamination is also possible. Transmission typically occurs via infected skin or hair cells. Additionally, dermatophytes may colonize the scalp of asymptomatic carriers, who can contribute to the spread of infection and maintenance of pathogens within the population [6]. To initiate infection, dermatophytes must overcome the skin’s natural barriers, particularly the stratum corneum. Conditions that compromise skin integrity, such as maceration or occlusion, increase susceptibility. Once these barriers are breached, fungi colonize the epidermis, beginning with the attachment of arthroconidia to keratinocytes via specialized proteins, which enables further development of the infection [7].

Zoophilic and geophilic dermatophytes typically result in acute skin infections characterized by pronounced inflammatory responses and intense pruritus [8]. Although most cases occur in resource-limited settings, dermatophytoses are globally distributed, with higher prevalence in Africa, Asia, and parts of southern and eastern Europe [9]. Contact with animals, especially rodents (guinea pigs, hamsters) and mammals (cats, dogs), may suggest fungal infection. Guinea pigs are often asymptomatic carriers of pathogens, which complicates the detection of infection. Differential diagnosis should include, among others, bacterial folliculitis, seborrheic dermatitis, cutaneous tuberculosis, psoriasis, alopecia areata, and scarring lesions following lupus erythematosus [10].

The most prevalent fungi causing SFIs among children are *Microsporum* spp., *Trichophyton* spp., and yeasts of the genus *Candida* spp., which under specific conditions can transition from colonization to a disease state.

*M. canis* is often responsible for SFIs, especially tinea capitis, a dermatophytic infection of the scalp. This dermatophyte grows on keratinized tissues such as skin, hair, and nails, and is a zoonosis mainly transmitted from cats to humans. Children, immunocompromised individuals, and those in contact with animals are particularly susceptible to infection. About 20% of cats can be asymptomatic carriers of *M. canis,* complicating infection control. In healthy children, fungal infections usually have a mild course, though immunosuppressed individuals face a higher risk of complications and recurrences [11].

Within the genus *Trichophyton*, closely related species are grouped into complexes, including the *T. mentagrophytes* and *T. rubrum* complexes. The *T. mentagrophytes* complex comprises four species: *T. mentagrophytes*, *T. interdigitale*, *T. tonsurans*, and *T. equinum.* These species are significant dermatological pathogens due to their zoonotic potential and broad range of superficial skin and appendage infections [12]. Accurate species identification is essential but challenging due to close phylogenetic relationships within the *T. mentagrophytes* complex [13].

*T. rubrum* is the most common cause of skin and nail infections, encompassing various forms of dermatophytosis. Infection primarily occurs through direct or indirect contact [14]. The fungus manifests as dry, ring-shaped, scaly skin lesions affecting the feet (tinea pedis), trunk (tinea corporis), groin (tinea cruris), and nails (onychomycosis). These lesions tend to have a relatively mild course and develop slowly [14,15]. *T. rubrum* adheres to epithelial cells through specific adhesins that recognize carbohydrates on the surface of microconidia. Subsequently, arthroconidia detect favorable environmental conditions, activating their metabolism and hyphal growth [16]. Virulent enzymes such as lipases, phospholipases, proteases, hemolysins, and elastases play key roles in infection development by facilitating fungal adhesion, growth, and tissue invasion [15].

*T. mentagrophytes* is a zoophilic dermatophyte, ranking second in prevalence after *T. rubrum*. This pathogen adheres to the host’s surface through fibrillar projections and specific surface proteins. Its infectious potential relies on a multi-step keratin degradation process, beginning with sulfitolysis—the hydrolysis of keratin’s disulfide bonds by enzymes cysteine dioxygenase and sulfite efflux pump. Following this, keratin is further broken down into peptides and amino acids by proteases, such as metalloproteinases (e.g., Mep4, Mep5) and subtilisins (e.g., Sub6). The ZafA gene plays a crucial role in zinc uptake, which supports fungal growth and reproduction [16]. *T. mentagrophytes* infects keratinized tissues of the skin, hair, and nails, causing common SFIs, known as dermatophytoses. Zoophilic strains particularly induce tinea capitis and tinea corporis with strong inflammatory responses. The pathogen can cause chronic and recurrent infections, with some isolates showing resistance to terbinafine and other antifungals, complicating treatment [16].

Another important etiological factor is *T. tonsurans*, which is the main cause of tinea gladiatorum, particularly among young athletes such as wrestlers. Infection is mainly transmitted through direct skin-to-skin contact, including from asymptomatic carriers [17]. *T. tonsurans* attacks the hair and hair follicles of the scalp, penetrating the interior of the hair shaft and causing weakening and breakage. A characteristic symptom of the infection is the presence of dark dots on the bald patch, known as “black dot” tinea [18].

*T. verrucosum*, on the other hand, mainly causes dermatophytosis in cattle. The infection spreads between animals or from the environment. In humans, especially children and farm workers, it causes skin lesions on the scalp and limbs. The disease mainly affects young cattle, worsens in winter, and leads to economic losses. The fungal infection is highly contagious and difficult to control due to the fungi’s prolonged survival in moist environments. Prevention requires hygiene, disinfection, and controlled farming conditions [19].

The main dermatophyte species and their typical features are presented in Table 1.

*Candida (C.) albicans* is a polymorphic fungus, a natural component of the human microbiome. It usually remains a harmless commensal, but can cause both superficial and systemic infections under favorable conditions. Its pathogenic traits include the ability to adhere to and invade host cells, secretion of hydrolytic enzymes, transition to filamentous forms, biofilm formation, phenotypic switching, and other adaptive mechanisms [20]. Candidalysin is a toxin secreted by invasive *C. albicans* strains that creates pores in cell membranes and activates epithelial cells. It stimulates signaling pathways (MAPK, NF-κB, PI3K), triggering an inflammatory response involving pro-inflammatory cytokines, neutrophil activation, and inflammasome engagement. While it increases fungal virulence, it simultaneously stimulates epithelial defense. The greatest damage occurs in immunocompromised individuals with high toxin levels [21,22]. *Candida* infections most commonly affect sites where mucous membranes and skin meet, such as the oral cavity, skin folds (groin, corners of the mouth), and the diaper area. Esophageal candidiasis is rarer, primarily occurring in infants up to one month old and immunocompromised individuals. Chronic and recurrent yeast infections, often associated with immune disorders, may also involve nails and require long-term treatment [23].

## 3. Epidemiology of Fungal Infections in Children

SFIs represent a widespread and clinically significant issue in the pediatric population, with a highly variable distribution influenced by geographic, environmental, socioeconomic, and host-related factors. These infections, which include dermatophytoses and candidiasis, are particularly prevalent in children aged 4–17 years, with tinea capitis being the most common manifestation in early childhood, especially among children aged 3–7 years. Tinea capitis is more frequently diagnosed in boys and in rural populations, likely due to higher exposure to animals, overcrowded living conditions, and limited access to hygiene and healthcare resources. In infants, candidal infections such as diaper dermatitis and oral thrush predominate, whereas adolescents tend to develop tinea pedis, onychomycosis, and tinea corporis, often due to increased physical activity, communal sports, and the use of shared facilities.

The dominant fungal species also differ by region and age group. In Central Europe, *Microsporum canis* is the primary cause of tinea capitis, particularly in southern countries as well as Austria, Germany and Hungary [24]. In contrast, anthropophilic species such as *T. tonsurans* and *T. soudanense* are increasingly reported across Europe, Israel, Argentina and Mexico, with rising cases attributed to factors such as migration from endemic regions, salon-based transmission, and changes in personal grooming habits [25,26,27]. In African countries such as Mauritania and Republic of Côte d’Ivoire, tinea capitis is endemic, with prevalence rates ranging from 10.5% to 23%, and is more common among males and rural populations [28,29]. The primary causative agents in these regions include both anthropophilic and zoophilic species from the *Trichophyton* and *Microsporum* genera. Local studies, such as those from Poland, demonstrate shifting patterns in fungal species, with historical dominance of *M. canis* giving way to increasing cases of *T. rubrum*, *T. tonsurans*, and *T. mentagrophytes*, particularly in infections of the trunk, limbs, nails, and scalp [30,31,32]. These changes reflect broader epidemiological trends driven by urbanization, globalization, increased mobility, and environmental shifts.

Key risk factors for superficial fungal infections include prolonged warm and humid climates, low socioeconomic status, overcrowding, poor hygiene, and close contact with infected individuals or animals [3]. Host-related factors such as young age, reduced sebum production, certain ethnic backgrounds, anemia, type 2 diabetes, immunosuppressive conditions (including leukemia, organ transplantation, and HIV/AIDS), and the use of immunosuppressive therapies also play a significant role in susceptibility. Despite the high burden of disease, preventive measures remain insufficient, and coordinated efforts across sectors are needed to improve early diagnosis, treatment, and public health education [2]. Enhanced epidemiological surveillance, especially in schools and high-risk communities, along with interdisciplinary collaboration among dermatologists, pediatricians, mycologists, and veterinarians, is essential to mitigate the spread and impact of these infections globally.

## 4. Clinical Manifestations

### 4.1. Tinea Capitis

Tinea capitis (fungal infection of the scalp) is a common dermatophyte infection affecting the scalp and hair follicles, mainly in children aged 3 to 7 years [33]. It is particularly prevalent among children of African American and Latino descent. The causative agents are dermatophytes from the *Trichophyton* and *Microsporum* genera, transmitted from human or animal sources [34]. Geographic variation in etiological agents is notable: *M. canis* is the most frequently isolated species in Europe and parts of Asia, while *T. tonsurans* predominates in North and South America as well as the United Kingdom [34,35].

**Figure 1:**
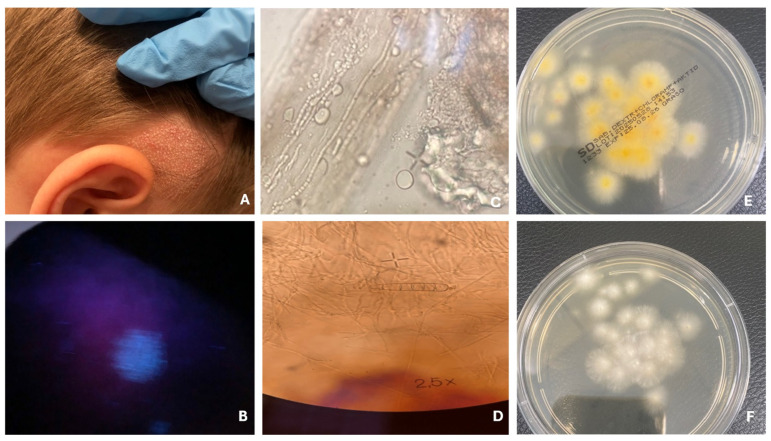
Tinea capitis—microsporic type. Patch hair loss (**A**). Fluorescence under Wood’s lamp (**B**). Fungal hyphae and spores on the hair shaft (**C**). Macroconidia of *M. canis* (**D**). Fungal culture on Sabouraud agar with chloramphenicol and Actidion, front side (**E**) and reverse side (**F**).

**Figure 2:**
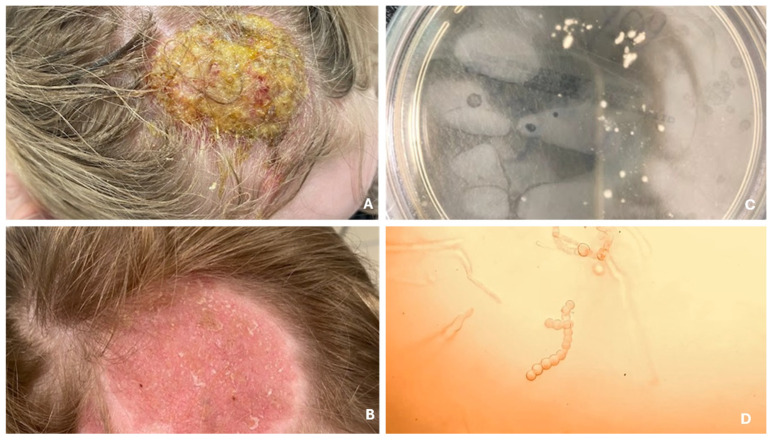
Infection of the scalp from cattle, presenting as Kerion celsi (**A**). Follow-up after several weeks of terbinafine treatment (**B**). Fungal culture on Sabouraud agar with chloramphenicol and Actidion (**C**). Hyphae with branching resembling deer antlers, indicating *T. verrucosum* (**D**).

There are four main clinical types of tinea capitis infections. Favus is the most severe form, leading to scarring and permanent hair loss. The trichophytic type features numerous small patches of hair loss and endothrix invasion (fungus inside the hair), while the microsporic type presents with larger patches and ectothrix invasion (fungus on the outer surface of the hair) (Figure 1) [36]. Kerion is an inflammatory form characterized by pustular folliculitis in a localized area of the scalp. It is a severe dermatosis, most commonly caused by *T. tonsurans*, *T. verrucosum*, and *T. mentagrophytes*, and less frequently by *M. canis*. The disease is characterized by intense inflammation, manifested by painful, red, and swollen lesions on the skin, which lead to damage of hair follicles and often result in scarring hair loss (Figure 2) [37]. A retrospective study conducted at Shahid Faghihi Hospital over 11 years is one of the largest epidemiological analyses in Iran. It showed that kerion occurs in both rural and urban areas, often associated with contact with animals, especially sheep [38]. Although mainly seen in children, it should also be considered in adults. Early diagnosis prevents complications such as skin scarring. Most patients experience hair regrowth, although with scarring. In severe cases, oral corticosteroids are recommended to control inflammation [38].

### 4.2. Tinea Corporis

Tinea corporis is a superficial skin infection affecting areas other than the hands, feet, face, scalp, groin, and nails. It is caused by dermatophytes from the genera *Trichophyton*, *Microsporum*, and *Epidermophyton*, transmitted by humans, animals, or contaminated objects. The disease is common, especially in tropical climates. Predisposing factors include moisture, heat, sharing towels, tight clothing, weakened immunity, and diabetes. It manifests as a red, scaly patch with a raised border and itching [39].

**Figure 3:**
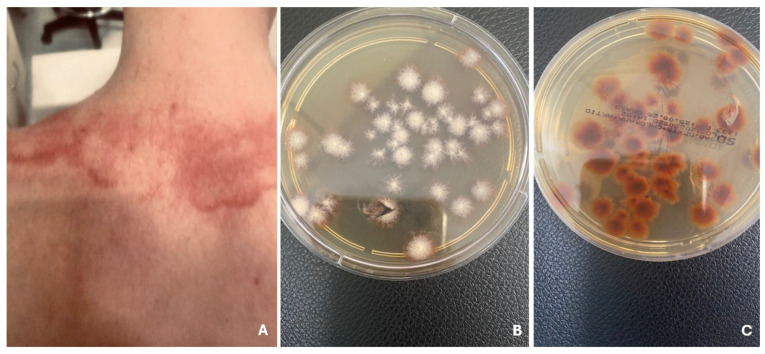
Well-demarcated, red plaques with a lighter center, in child practicing martial arts (**A**). Tinea corporis gladiatorum. *T. tonsurans* on Sabouraud agar with chloramphenicol and Actidion, front side (**B**) and reverse side (**C**).

Tinea gladiatorum is a variety of tinea corporis that appears as well-demarcated, red, scaly papules and plaques with a lighter center, most commonly on the head, neck, and upper limbs—areas of contact during wrestling (Figure 3). It is commonly seen in contact sports athletes, spreading mainly through skin-to-skin contact and indirectly via contaminated items such as training mats [27]. Symptoms may include itching, redness, burning, and scaling; however, many individuals are asymptomatic at the time of medical evaluation [40].

### 4.3. Tinea Pedis

**Figure 4:**
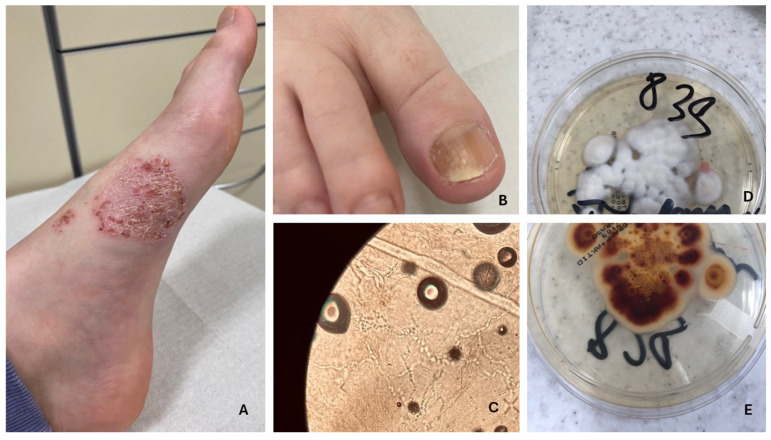
Tinea pedis and onychomycosis. (**A**,**B**) Fungal spores present in the KOH preparation (**C**). Fungal culture on Sabouraud agar with chloramphenicol and Actidion—*T. rubrum*, reverse side (**D**), and front side (**E**).

Tinea pedis, commonly known as athlete’s foot, occurs frequently in adolescents and is generally rare in younger children [41]. The development of the infection is promoted by wearing closed footwear, which creates a warm, moist environment conducive to fungal growth [41,42]. Transmission often occurs through contaminated floors in communal areas such as changing rooms, swimming pools, and shared household spaces [41]. Although tinea pedis is less studied in the pediatric population, its prevalence is higher in warm, humid climates, and the risk increases with age [42]. Additional contributing factors include walking barefoot, wearing sandals, and using shared bathrooms [42]. The most prevalent causative agents are dermatophytes, particularly *T. rubrum*, followed by *T. mentagrophytes*, *E.floccosum*, and, in pediatric cases, *T. tonsurans* [41,42]. Clinically, four types of tinea pedis are recognized: interdigital, the most common form, characterized by redness and scaling between the toes; ulcerative, presenting with erosions and ulcers; moccasin type, involving diffuse redness and scaling of the soles; and inflammatory, which features blisters and pustules and is often associated with zoophilic fungi (Figure 4) [42]. Early diagnosis and treatment are essential to prevent complications and chronic infection [42]. However, a lack of standardized diagnostic procedures complicates accurate assessment of prevalence in children, highlighting the need for increased awareness among healthcare providers and caregivers [42].

### 4.4. Onychomycosis

Onychomycosis, although less common in children than in adults, is increasingly being recognized as a relevant pediatric condition, with prevalence rates reported between 0.14% and 7.66% depending on the population studied and diagnostic criteria used [23,43,44]. While culture-confirmed global prevalence remains relatively low at approximately 0.33%, recent studies suggest a gradual, albeit statistically insignificant, rise in pediatric cases over recent decades [44,45]. The infection commonly affects children between the ages of 6 and 11, particularly school-aged children, who are more susceptible due to factors such as increased sweating, nail trauma, and the frequent use of closed footwear [23,46]. Dermatophytes, especially *T. rubrum* and *T. mentagrophytes*, are the leading causative agents, although non-dermatophyte molds like *Fusarium* are responsible for about 17% of cases, with their frequency increasing with age [23,45,46]. In younger children, particularly those under six years old, yeasts may more commonly affect fingernails [23]. The most prevalent clinical form is distal and lateral subungual onychomycosis (Figure 4), followed by the superficial white type, which appears more frequently in pediatric patients compared to adults [23,43]. Onychomycosis is often associated with tinea pedis, and studies indicate that approximately one-quarter of children with nail infections also present with athlete’s foot [43]. Although still underdiagnosed in this age group, onychomycosis should be considered in the differential diagnosis of nail dystrophies, and confirmation via mycological testing is essential to guide appropriate treatment [43]. Fusarium infections are particularly challenging due to their tendency to recur and their resistance to standard therapies, making topical treatment the preferred first-line approach in many pediatric cases [46]. Early diagnosis and targeted therapy are crucial for effective management, preventing complications, and reducing the likelihood of chronic or recurrent infections.

### 4.5. Candidiasis

**Figure 5:**
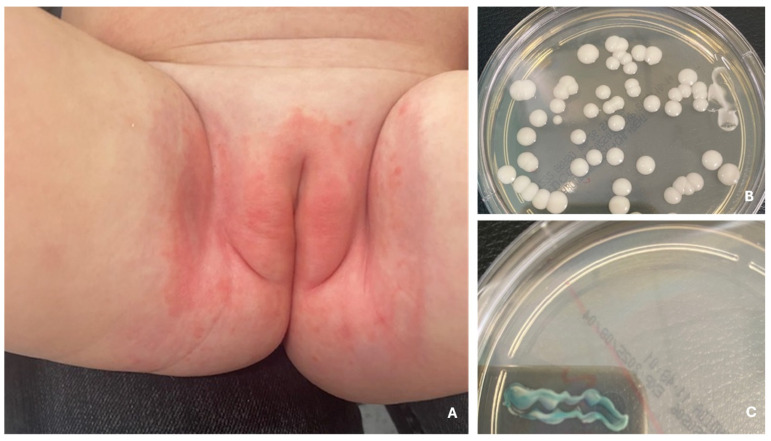
Redness, swelling and maceration in the diaper area—diaper candidiasis caused by *C. albicans* (**A**). Diaper candidiasis. Colonies of *C. albicans* after 24 h of incubation at 37 °C (**B**), green coloration of colonies on Chromagar *Candida* medium after 24 h of incubation at 37 °C (**C**).

**Figure 6:**
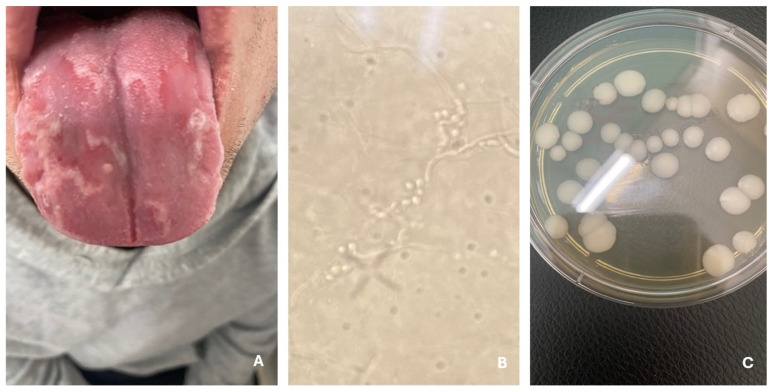
Oral candidiasis (**A**). Hyphae and yeasts (**B**). *C. albicans*—fungal culture on Sabouraud agar with chloramphenicol and gentamicin (**C**).

Candidiasis is a common fungal infection in children, primarily caused by *Candida albicans*, a species that frequently colonizes the skin and mucous membranes even in healthy individuals [47]. Despite the presence of protective immune mechanisms, candidiasis can develop, particularly in areas of increased moisture, such as the diaper region in infants, where elevated skin pH and the presence of fecal enzymes create an ideal environment for fungal growth [47]. Diaper candidiasis is especially prevalent in newborns and infants, triggered by diarrhea in about one-third of cases [48]. It typically presents as redness, swelling, maceration, and satellite pustules confined to the diaper area but may spread to or progress to intertrigo, affecting other skin folds [48]. In more severe cases, ulcers and erosions may form, causing discomfort, pain, or itching (Figure 5) [48]. Children with compromised immune function, skin conditions like atopic dermatitis or psoriasis, or those using immunosuppressive medications are at increased risk of cutaneous candidiasis [47]. Oral candidiasis is another common manifestation, appearing acutely or chronically, usually as painful, erythematous, or white pseudomembranous lesions on the mucosa, which may extend to the larynx or pharynx, leading to difficulty eating and swallowing (Figure 6) [49,50]. Forms such as angular stomatitis (characterized by cracks and redness at the corners of the mouth) and hyperplastic candidiasis (with thickened mucosal lesions) can also occur [50]. Oral thrush often precedes systemic or invasive candidiasis and is most common in young children with immature or suppressed immune systems, dry mouth, or those using corticosteroids [50]. Candidiasis may also involve the nails, genital area, and other skin regions [50]. In rare cases, chronic mucocutaneous candidiasis (CMC) develops in children with underlying immunodeficiencies and is marked by persistent, recurrent fungal infections affecting the skin, nails, and mucosa [50].

### 4.6. Tinea Incognito

Tinea incognito (TI) was first described in 1968 as an altered form of skin fungal infection caused by improper use of corticosteroids and calcineurin inhibitors [51]. Easy access to steroids contributes to the development of TI—with 15.5% of patients using them before dermatological consultation. Steroids weaken the skin, increasing the risk of injury and infection [52]. The prevalence of TI is estimated to be 5.6–35% in children and 3.8–5.6% in adults, with the number of cases increasing [51]. Atypical clinical presentation complicates diagnosis, delaying treatment and increasing therapy costs. Diagnosis requires vigilance and additional tests [52]. The problem affects various specialists, making correct diagnosis and treatment essential [51].

### 4.7. Emycetoma

Mycetoma is more than just a chronic skin infection caused by fungi (eumycetoma) or bacteria (actinomycetoma), commonly found in tropical regions—it also poses serious social and educational challenges [53]. In 2024, the World Health Organization (WHO) recognized eumycetoma in children, caused by over 60 fungal species, as a high-priority disease [54]. While the foot is the most frequently affected site, leading to swelling, sinus formation, and purulent discharge, the consequences extend far beyond physical health. Infected children often struggle with everyday activities, learning, and social inclusion [54]. A review of data from nearly 6800 patients treated in Khartoum between 1991 and 2014 reveals that eumycetoma primarily affects young boys, particularly students, with the foot and hand being the most common sites of infection [55]. Less commonly, the disease affects areas such as the leg, knee, thigh, buttocks, and arms [55]. Diagnosis typically relies on histopathology, cytology, and ultrasonography. Treatment involves a combination of antifungal and antibacterial medications, often alongside surgery. However, recurrence occurs in nearly 18% of pediatric cases, further complicating prognosis and quality of life [53]. The most frequently identified causative agents include *Madurella mycetomatis*, *Falciformispora senegalensis*, *Trematosphaeria grisea*, *Scedosporium boydii*, and *Medicopsis romeroi* [54]. Ultimately, eumycetoma in children is not only a medical concern but also a significant social and educational issue, affecting many aspects of young patients’ lives beyond the visible symptoms [55].

## 5. Identification

Accurate and timely diagnosis of SFIs in children is essential for effective treatment and prevention of complications or transmission. While traditional methods remain the foundation of dermatological diagnostics, modern molecular techniques are increasingly integrated into clinical practice due to their enhanced sensitivity, specificity, and speed. Diagnostic strategies must be adapted to the patient’s age, clinical presentation, infection site, and potential exposure history (e.g., contact with animals or shared environments).

The conventional approach to diagnosing dermatophytoses primarily relies on direct microscopic examination (DME) of clinical material, fungal culture, and morphological identification of isolated pathogens [56,57,58]. DME, often performed using potassium hydroxide (KOH) preparations, enables the visualization of fungal hyphae or spores directly from skin scrapings, hair, or nail samples [56,59]. Although rapid and inexpensive, the sensitivity of this method is limited, and it does not allow for species-level identification [56,60]. 

Fungal culture remains the gold standard for confirming infestation and identifying the causative organism [57]. Samples are typically grown on selective media such as Sabouraud dextrose agar or potato dextrose agar, often supplemented with antibiotics (e.g., chloramphenicol) and cycloheximide to inhibit bacterial and saprophytic growth [57]. However, fungal cultures are time-consuming, requiring 1 to 5 weeks for results, and their accuracy depends heavily on proper specimen collection, laboratory conditions, and the expertise of personnel [56,59,60]. Infections may be missed if prior antifungal or corticosteroid therapy has modified the lesion or suppressed fungal growth [60]. Despite these limitations, culture remains critical for species identification and susceptibility testing in difficult or treatment-resistant cases [60]. For yeast infections, such as those caused by *Candida* species, microscopic evaluation is followed by subculture on chromogenic media, which facilitates preliminary identification based on colony color and morphology [61]. For example, *C. albicans* produces green colonies, *C. tropicalis* metallic blue, and *C. krusei* pink, fuzzy colonies [61]. If species-level identification is not possible using chromogenic or biochemical profiles, isolates are classified broadly as *Candida* spp [61].

The Wood’s lamp is a simple, non-invasive diagnostic tool that has regained popularity in recent years due to its affordability and ease of use, particularly in primary care and pediatric settings [21,62]. It emits ultraviolet light, allowing certain fungal species to fluoresce under specific wavelengths [21,62]. For example, infections caused by *M. canis* may exhibit bright green fluorescence, while favus (caused by *T. schoenleinii*) shows dull green fluorescence [59]. Conversely, *Trichophyton* infections often do not fluoresce, which limits the sensitivity of this method [59]. Nevertheless, the Wood’s lamp is especially helpful in identifying the most appropriate area for sampling and can assist in early diagnosis, particularly in tinea capitis [63]. Its utility is heightened when used in conjunction with a thorough clinical history, including possible animal contact.

Biochemical tests, such as the urease test and hair perforation test, are used to confirm the presence of fungal species. The urease test is performed on Christensen’s agar medium, where urease-positive organisms cause the medium to change color from yellow to red [58]. *T. mentagrophytes*, *M. gypseum*, and *E. floccosum* show urease activity, while *T. rubrum* is urease-negative. The hair perforation test involved inoculating a sterile hair fragment with the fungus and incubating it for four weeks; a positive result, indicated by wedge-shaped damage on the hair, was observed for *T. mentagrophytes* and *M. gypseum*, whereas *E. floccosum* and *T. rubrum* showed a negative result [64,65].

In atypical presentations, deep lesions or when there are underlying conditions that obscure typical signs of infection, skin biopsy with histopathological examination becomes valuable [22]. This method allows for assessment of tissue architecture, identification of inflammatory patterns, and direct visualization of fungal elements using special stains [22]. It can support diagnosis even before culture results are available and is particularly useful in immunocompromised patients or when invasive mycoses are suspected [22].

Polymerase chain reaction (PCR)-based methods are increasingly utilized in the diagnosis of dermatomycoses due to their superior sensitivity, specificity, and rapid turnaround time compared to conventional techniques [58,60]. PCR allows for direct detection of fungal DNA in clinical specimens, bypassing the need for culture [66,67,68]. Both conventional and real-time PCR (RT-PCR) are used, with protocols targeting various genetic regions (e.g., internal transcribed spacer (ITS), 28S rDNA, topoisomerase II genes, chitin synthase I) [66]. These assays are especially useful in identifying pathogens from samples with low fungal load or in cases where traditional culture yields negative results despite clinical suspicion [66]. RT-PCR is particularly effective in diagnosing infections caused by *Microsporum canis*, a common dermatophyte in children. Commercial PCR kits offer user-friendly DNA isolation and are increasingly being adopted in clinical laboratories. However, a limitation of these kits is their often narrow target range, as many are designed to detect only the most common species [69]. Consequently, rare or emerging pathogens may be missed unless broad-range or sequencing-based PCR approaches are employed [69].

An advanced tool gaining prominence in medical mycology is Matrix-Assisted Laser Desorption/Ionization Time-of-Flight Mass Spectrometry (MALDI-TOF MS). This technique analyzes protein spectra from fungal isolates and compares them with a reference database for precise species identification [70,71]. MALDI-TOF offers high accuracy—studies show it can correctly identify *T. mentagrophytes* in nearly 90% of cases and has the advantage of being rapid, cost-effective, and user-friendly once a database is established [70,71]. Unlike PCR, it does not require DNA extraction or amplification and overcomes limitations of both traditional culture and expensive molecular diagnostics [70,71]. Its growing adoption is replacing older biochemical and morphological methods.

In some cases, imaging diagnostic methods are also utilized. Ultrasound imaging is useful in diagnosing onychomadesis and retronychia by clearly showing defects beneath the proximal nail fold. It can also help exclude other conditions like abscesses or subungual tumors. In onychomadesis, two nail plate fragments—proximal and distal—are typically visible, and ultrasound can help estimate when the damage occurred. In retronychia, it often reveals a thickened nail plate under the proximal nail fold [72].

Another non-invasive tool that can also be implemented in dermatophyte detection is reflectance confocal microscopy (RCM). This in vivo imaging method provides real-time visualization of the skin. In cases of tinea, RCM typically reveals bright, linear, branching filamentous structures within the stratum corneum. Although not widely used, RCM shows promise as a highly sensitive and potentially effective tool for diagnosing cutaneous dermatophyte infections [73,74].

Despite advances in diagnostic methods, several challenges remain in identifying superficial fungal infections, especially in pediatric populations. Misdiagnosis can occur due to prior use of topical corticosteroids or antifungals that alter lesion appearance, leading to conditions like tinea incognito. Comorbidities and atypical immune responses may also obscure classic signs, particularly in infants or immunocompromised children. Therefore, clinicians must maintain a high index of suspicion and use a combination of clinical examination and laboratory tools to reach a definitive diagnosis.

Rapid and accurate identification of the causative agent not only facilitates appropriate treatment selection but also reduces patient discomfort, limits unnecessary therapy, and prevents further spread, which is particularly important in school and daycare settings. In all cases, effective diagnosis should be supported by proper specimen collection, awareness of risk factors (e.g., animal exposure, shared facilities), and the integration of modern tools such as PCR, Wood’s lamp, or MALDI-TOF into clinical practice where available.

## 6. Treatment

The treatment of superficial fungal infections in children depends on several key factors, including the type of fungal pathogen, the site and severity of the infection, and individual patient characteristics such as age, immune status, and comorbidities [75]. Topical antifungal agents are the first-line therapy for most uncomplicated infections, particularly those affecting the glabrous skin, such as tinea corporis, tinea cruris, and tinea pedis [76]. Commonly used topical drugs include imidazoles (e.g., clotrimazole, ketoconazole, miconazole), allylamines (e.g., terbinafine, butenafine), and other agents like ciclopirox olamine [75,77]. Among these, allylamines like terbinafine and butenafine are noted for their rapid and potent antifungal activity, often resulting in shorter treatment durations and higher efficacy compared to imidazoles, which have broader antifungal spectra but slower action [75,76]. Ciclopirox, although less commonly used, remains effective against a wide range of superficial mycoses. It is important to note that nystatin, though useful in treating Candida infections, is ineffective against dermatophytes and should not be used for infections such as tinea corporis or tinea pedis [76].

Systemic antifungal therapy is generally reserved for cases where topical agents cannot adequately penetrate the affected tissue or when the infection is widespread. This is particularly the case with tinea capitis, where oral antifungals are necessary due to the involvement of hair follicles, onychomycosis, which affects the nails and often requires prolonged treatment and tinea incognito, where systemic therapy is necessary to address the often atypical and more severe presentation [76]. Oral antifungal agents commonly used in children include terbinafine and itraconazole, both of which have proven effective in treating dermatophyte infections. Terbinafine, in particular, is regarded as the drug of choice for dermatophytoses caused by *Trichophyton* species due to its fungicidal mechanism of action—specifically the inhibition of squalene epoxidase, an enzyme essential for ergosterol synthesis in fungal cell membranes [78]. This disruption leads to the accumulation of toxic squalene and ultimately fungal cell death [78]. Terbinafine is favored not only for its high clinical efficacy but also for its excellent pharmacokinetic profile, low incidence of adverse effects, and minimal risk of drug interactions [78]. Compared to older agents like griseofulvin, terbinafine demonstrates significantly lower relapse rates in the treatment of onychomycosis—39% versus 76%, respectively—making it a superior choice in both pediatric and adult populations [78]. However, resistance to terbinafine is becoming an increasing concern, particularly with *T. rubrum* and *T. indotineae*, strains associated with mutations in the SQLE (squalene epoxidase) gene [78,79]. These resistant strains have been especially problematic in India and have also been reported in other countries such as Iran [78,79]. To address this challenge, combination therapy, typically involving oral terbinafine or itraconazole together with a topical azole, is often recommended [78,80]. This strategy enhances antifungal efficacy and reduces the risk of resistance development [81]. It is also advisable to extend treatment by an additional 2–4 weeks in cases where clinical symptoms improve but mycological testing remains positive [80]. Nail infections (onychomycosis), which are notoriously difficult to treat, may require systemic therapy lasting several months, with careful monitoring to ensure adherence and manage potential side effects [76]. While topical therapies alone are insufficient for nail infections, newer FDA-approved topical agents such as efinaconazole, tavaborole, and ciclopirox have been introduced, though their use in children remains limited and often adjunctive. In scalp infections like tinea capitis, where systemic treatment is essential, adjunctive use of antifungal shampoos containing ketoconazole or selenium sulfide during the initial 1–2 weeks of therapy can help decrease fungal load and prevent transmission to others, particularly in school settings [76]. Summary of systemic therapies of SFIs is provided in Table 2.

In summary, the treatment of SFIs in children depends on the type of fungus, infection site, and individual patient factors. Topical antifungals are mainly used, with oral therapy reserved for more severe cases. Terbinafine is the drug of choice due to its effectiveness and safety. Combining therapies reduces resistance risk and improves treatment outcomes. Treatment duration ranges from weeks to months, with patient monitoring being important. Resistance is increasing, especially in *Trichophyton* spp., but terbinafine remains effective. Antifungal shampoos are used early in scalp infections, while systemic therapy is needed for tinea incognito and nail infections.

## 7. Conclusions

Superficial fungal infections of the skin, nails, and hair, primarily caused by dermatophytes (e.g., *Trichophyton*, *Microsporum*) and yeasts (*Candida*), represent a growing health problem, especially in children. Infections are transmitted through contact with infected individuals, animals, or contaminated environments. Factors predisposing children to infections include immature immune systems, poor hygiene, and contact with animals. In children, the most common infection is tinea capitis (scalp dermatophytosis), which may manifest in various forms, including the inflammatory kerion. Diagnosis requires microscopic examination and culture, while treatment mainly relies on terbinafine, although resistant pathogens and relapse risks are emerging. Effective diagnosis and therapy are key to limiting the spread of fungal infections and preventing complications.

## Figures and Tables

**Table 1:** Primary dermatophyte species and their typical characteristics [14].

Ecological Habitat	Dermatophyte Species	Typical Clinical Manifestations	Geographic Range/ Epidemiology
Anthropophilic (human)	*T. rubrum*, *T. interdigitale*, *T. tonsurans*	Tinea pedis, corporis, onychomycosis, capitis	Global
	*T. violaceum*, *M. audouinii*, *E. floccosum*	Tinea pedis, capitis, onychomycosis	Africa, global
Zoophilic (animal)	*T. mentagrophyte*, *M. canis*, *T. benhamiae*	Tinea corporis, capitis, cruris	Europe, Asia, USA, Japan
Geophilic (soil)	*Nannizzia gypsea*	Tinea corporis, capitis	Rare infections in humans

**Table 2:** Dosages and duration of treatment with systemic antifungal drugs in dermatophyte infections [72].

Disease	Fluconazole	Griseofulvin	Itraconazole	Terbinafine
Tinea capitis	Children: 6 mg/kg/day for 3–6 weeks Adults: 150–450 mg weekly	Children: 10–15 mg/kg/day (ultramicronized) or 20–25 mg/kg/day (micronized suspension) for 6–8 weeks Adults: 500 mg daily	Children: 5 mg/kg/day for 4–8 weeks Adults: 200 mg daily	Adults: 250 mg/day for 3–4 weeks Children (granules): <25 kg—125 mg, 25–35 kg—187.5 mg, >35 kg—250 mg for 3–4 weeks
Tinea corporis/cruris	Children: 6 mg/kg weekly (from general dose) Adults: 150–450 mg weekly (standard)	Children: 15–20 mg/kg daily (micronized) or 10–15 mg/kg daily (ultramicronized) for 2–4 weeks Adults: 500 mg daily	Children: 3–5 mg/kg daily for 1 week Adults: 200 mg daily for 1 week	Children: individually determined; usually 250 mg daily Adults: 250 mg daily for 1 week
Tinea unguium	Children: 6 mg/kg weekly (general Fluconazole dosing) Adults: 150–450 mg weekly	Children: 1–2 g/day (micronized) or 750 mg/day (ultramicronized) until healthy nail regrowth Adults: 500 mg daily	Children: 200 mg daily for 12 weeks or 200 mg BID one week/month for 2–4 months Adults: 200 mg daily	Children: individually determined; usually 250 mg daily Adults: 250 mg daily for 12 weeks
Tinea pedis	Children: 6 mg/kg weekly (general dose) Adults: 150–450 mg weekly	Children: 15–20 mg/kg daily (micronized) or 10–15 mg/kg daily (ultramicronized) for 4 weeks Adults: 500 mg daily	Children: 3–5 mg/kg daily for 1 week Adults: 200 mg daily for 2 weeks	Children: individually determined; usually 250 mg daily Adults: 250 mg daily for 2 weeks

## Data Availability

No new data were created or analyzed in this study. Data sharing is not applicable to this article.

## Abbreviations

The following abbreviations are used in this manuscript:

AIDS	Acquired Immunodeficiency Syndrome
CMC	Chronic Mucocutaneous Candidiasis
DME	Direct Microscopic Examination
DMSO	Dimethyl Sulfoxide
DNA	Deoxyribonucleic Acid
HIV	Human Immunodeficiency Virus
KOH	Potassium Hydroxide
MALDI-TOF MS	Matrix-Assisted Laser Desorption/Ionization Time-of-Flight Mass Spectrometry
MAPK	Mitogen-Activated Protein Kinase
NF-κB	Nuclear Factor of kappa Light Chain Enhancer of Activated B Cells
PCR	Polymerase Chain Reaction
PI3K	Phosphoinositide 3-kinase
RCM	Reflectance Confocal Microscopy
RT-PCR	Real-Time Polymerase Chain Reaction
ITS	Internal Transcribed Spacer (ITS)
SFIs	Superficial Fungal Infections
WHO	World Health Organisation
28s rDNA	28S Ribosomal DNA
